# Can follow-up reminders from postal questionnaires help determine the mechanism of missing data in trials?

**DOI:** 10.1186/1745-6215-14-S1-P108

**Published:** 2013-11-29

**Authors:** Ivana Holloway, Amanda Farrin, Anne Forster

**Affiliations:** 1 University of Leeds, Leeds, UK; 2 Bradford Institute for Health Research, Bradford, UK

## 

The issue of missing data is present in every trial. Identification of the missing data mechanism is not straightforward; however it is essential to avoid bias. The mechanism of missing completely at random (MCAR) is seldom appropriate and the distinction between missing at random (MAR) and not at random (MNAR) is not straightforward. Follow-up reminder responses provide excellent source of information for investigating the issue.

Improving postal response rates for patient-reported outcome measures often requires some kind of reminder process. Patient-reported outcomes from two stroke rehabilitation cluster randomised trials were used to investigate the usefulness of reminders in the identification of the missing data mechanism. In both trials, the reminder process was set-up from the outset. Missing data in these trials had an intermittent pattern. An approach by Fairclough (2010) was used to determine missingness mechanism.

Covariates predicting non-response (from reminder-responders) were first identified to distinguish between MCAR and MAR mechanisms and participants outcome scores were added to model testing for inclusion to check for evidence of MNAR.

The advantage of this method is that reminder-responses are considered to be similar to non-responders with the additional benefit of knowing the actual outcome. Furthermore, identification of covariates indicated by this approach can inform the choice of covariates for the appropriate method of data imputation.

In trials with potentially high loss to follow-up, reminder strategies can be used not only to minimise loss to follow-up but offer valuable data to examine mechanisms of missingness and ultimately contribute to a robust final analysis.

